# The effect of the menstrual cycle phases on back squat performance, jumping ability and psychological state in women according to their level of performance -a randomized three-arm crossover study

**DOI:** 10.1186/s13102-024-01010-4

**Published:** 2024-11-02

**Authors:** Eduard Isenmann, Steffen Held, Stephan Geisler, Ulrich Flenker, Ian Jeffreys, Christoph Zinner

**Affiliations:** 1 Department of Fitness and Health, IST University of Applied Sciences, North Rhine-Westphalia, 40233 Dusseldorf, Germany; 2 Department of Molecular and Cellular Sport Medicine, Institute of Cardiology and Sports Medicine, German Sport University Cologne, North Rhine-Westphalia, 50933 Cologne, Germany; 3 Department of Sport and Management, IST University of Applied Sciences, 40233 Duesseldorf, North Rhine-Westphalia Germany; 4 Department of Intervention Research in Exercise Training, German Sport University Cologne, 50933 Cologne, Germany; 5 Setanta College, Thurles, E41 AP65 Ireland; 6 Department of Sport, University of Applied Sciences for Police and Administration of Hesse, 65199 Wiesbaden, Hesse Germany

**Keywords:** Female athletes, Squat, Performance, jumping ability, Menstrual cycle, Well-being

## Abstract

**Objective:**

The influence of the menstrual cycle on practical power performance such as barbell back squats and jumping performance in women has not yet been fully investigated. In addition, the performance level of athletes has not been considered in previous studies. This study aimed to investigate the influence of different cycle phases on acute back squat performance, jumping ability and psychological state concerning the performance level.

**Methods:**

24 female strength athletes (age: 25.2 ± 3.3 years; height: 169.5 ± 3.4 cm; body weight: 67.7 ± 7.3 kg) were recruited for the study. Level of performance was classified according to Santos et al. (intermittent (*n* = 13), advanced (*n* = 6), highly advanced (*n* = 5)). Participants were tested for 1RM barbell back squat and jumping performance (countermovement and squat jump) as well as two questionnaires assessing their psychological states in the menses (M), late follicular phase (FP) and mid-luteal phase (LP) in three MC. Saliva estradiol and progesterone concentrations with a menstrual cycle diary were used to confirm a normal MC. A principal components analysis for power performance, well-being, relaxation and alertness was carried out and a linear mixed model was used for statistical evaluation.

**Results:**

No significant differences were found between the MC phases in performance scores (*p* > 0.05), readiness (*p* > 0.05) and alertness (*p* > 0.05). However, a high correlation between MC phases, performance level and back squat performance was detected. Correlation analyses indicate that a higher performance level results in a higher variation depending on the MC of the squat performance. For well-being, a significantly lower score was detected in M than in FP and LP.

**Conclusion:**

In general the performance score of the lower body is not influenced by the MC. If strength performance and jumping ability are considered separately, there are indications that strength capability is influenced at a higher performance level. In addition, individual variance was also observed, so this should also be considered. However, further studies are needed to confirm this assumption due to the small sample sizes of the individual performance levels.

**Trial registration:**

German registry for clinical studies (DRKS00034816, Date: 08/01/2024).

**Supplementary Information:**

The online version contains supplementary material available at 10.1186/s13102-024-01010-4.

## Introduction

Females remain an understudied population group in sport science [1]. In recent years, numerous investigations have focused on examining the impact of the menstrual cycle on exercise and sports performance. Estrogen and progesterone primarily regulate fertility in women, however, research indicates that these hormones can affect various functions within the body and, consequently performance [2–7]. The fluctuating concentrations of hormones during the menstrual cycle, particularly estrogen and progesterone could potentially impact performance, which has resulted in suggestions that strength performance and jumping ability could be affected at different stages of the cycle [8–10]. Estrogen influences actin-myosin binding potentially affecting muscle contraction capacity and thus maximal strength. On the other hand, progesterone may inhibit neural activity, acting as a counterbalance to estrogen [11]. Consequently, it is possible that as the relative influence of these hormones changes during the menstrual cycle, this can affect strength performance and jumping ability.

Some studies have found no significant fluctuations in strength performance throughout the menstrual cycle [12–19] while others have observed increased muscle strength shortly before ovulation [20–22]. Some of these diverging results could be the result of different testing methodologies. Most research in this area has focused on isometric strength which has only limited applicability to fast activities and sports disciplines [10]. Besides, observations about jumping ability are also inconsistent. For example, Julian et al. reported unclear results for CMJ performance between the early follicular and mid-luteal phases of the menstrual cycle [23]. These results are supported as no changes in CMJ performance depending on the menstrual cycle were observed neither in female handball players nor in physically active women [24, 25]. In contrast, Garcia-Pinillos observed a significantly higher performance in the follicular phase in the squat jump [26]. Potentially there are only trivial to small effects of MC on the strength-related measures, but these observations are not significant [27].

The divergent outcomes of these studies appear to stem from differences in study quality and the methods used to track menstrual cycle phases [8]. Another potential factor contributing to the varying results is the disparity in training status among the participants. Most studies have involved healthy but untrained or recreational females and as a result, little is known about the effects on trained athletes [8, 10, 28]. However, it is known that at higher performance levels, other factors such as well-being, sleep or non-adequate energy intake could play a role in maximum performance [29–31]. As a result, a comparison between trained women and untrained women, using the same methodology, could yield valuable insights as trained female athletes may experience different performance fluctuations during their menstrual cycle compared to untrained women. In addition to physical performance, the influence of different phases of MC on psychological aspects such as mood or well-being is also being investigated. Bancroft’s initial studies showed that well-being changes during the menstrual cycle [32]. Premenstrual symptoms can occur in women, especially before menstruation. In addition, self-reported mood, cognitive and physical symptoms are lower during menstruation compared to the other phases of the MC [33–35].

Even though there are already a few studies on the individual components, the correlation between MC and performance has not yet been explicitly analysed as a dependence on the level of performance. Therefore, this study aims to provide additional insights into the variations in maximal performance in back squats, jumping ability and psychological state during the menstrual cycle of women with different performance levels.

## Materials and methods

### Participants

A total of 24 young women (age: 25.2 ± 3.3 years; height: 169.5 ± 3.4 cm; body weight: 67.3 ± 7.3 kg) successfully participated in the study. The participants had to fulfil the following inclusion criteria.

1. Be pre-menopausal and be in the age range of 18–35 years.
2. Not being pregnant.
3. Not using hormonal contraceptives.
4. Have a regular menstrual cycle of 21–35 days and have been monitored for this for at least three months.
5. Be able to perform a squat following NSCA guidelines [36].
6. Have no injuries or illnesses that prevent them from completing the study.

Use of hormonal contraceptives, and lower limb injuries in the last 6 months excluded individuals from participation. All included participants could also be classified according to Santos [37] as intermediate (*n* = 13), advanced (*n* = 6) or highly advanced (*n* = 5) strength athletes. The study was conducted according to the guidelines of the Declaration of Helsinki and CONSORT guidelines. The study was approved by the ethics of IST University of Management, Dusseldorf, Germany (No. 092021IST233), approved in December 2021) and has been registered in the German registry for clinical studies (DRKS00034816, Date: 08/01/2024). All subjects gave written consent to participate study before the start of the study.

### Study design and procedure

A three-arm randomised study with a crossover design was conducted to examine the effects of the menses (M), late follicular phase (LFP) and mid-luteal phase (MLP) on maximum strength and jumping performance. In addition, questionnaires were used to assess well-being, relaxation state and alertness. Before the experimental investigation, the test persons performed two sessions of familiarisation for performance parameters to prevent learning and adaptation effects. The three assessment days for the performance tests took place over three months. There was a washout period of at least 3 weeks between the familiarisation phase and the first intervention and between each intervention day.

The examinations were usually conducted on one day of the different cycle phases: M: day 2–3, LFP: day 8–10 and MLP: day 20–23, depending on the individual cycle and for personal reasons of feasibility. The order of the cycle phases was individual and random for each athlete, as long as the washout phase was taken into account.

The subjects were instructed to eat a standardised breakfast on examination days, no later than 1 h before the start of the measurement. The breakfast contained one gram of oatmeal per kilogram body weight, one banana and 5 g of honey. The breakfast and the timing of the of meal and measurement were replicated for each visit to control for diurnal variations in corticospinal excitability and maximum force production. The participants were also instructed not to consume alcohol for at least 24 h, and caffeine for at least 12 h, before measurement. Intense physical activity was also prohibited for 48 h, and low physical activity for 24 h, before the performance tests.

### Monitoring menstrual cycle

The menstrual cycle was monitored by the participants using a cycle diary for at least three months before the assessment days. The beginning of a cycle was defined by each woman with the first day of bleeding. Based on this data, the future cycles were calculated and an initial date for the tests was set. With the start of a new cycle (day 1 of M), the study director was informed that an exact date (M, LFP, or MLP) for the examination day was set. In addition to the cycle diary, saliva samples were used to check estradiol and progesterone concentrations. Saliva analysis is a valid method to identify normal from abnormal hormonal cycles [38, 39]. The hormone concentrations were used exclusively to confirm a normal MC and the cycle phase, and not to determine absolute values. Since, the exact ovulation phase could not be measured, this phase was considered as five instead of the usual three days. In Table 1, documentation and planning of the intervention days of a participant with a cycle length of 28–32 days and a menses of 5 days are shown.

**Table 1:** Documentation and planning of the monitoring phase and the intervention phase of a participant with a cycle length of 28–32 days and a menses of 5 days

	Monitoring phase				Intervention phase		
Day	Month 1	Month 2	Month 3	Month 4	Month 5	Month 6	Month 7
1.		FP (12)	FP (12)	FP (13)	FP (13)	TO (14)	TO (15)
2.		FP (13)	FP (13)	TO (14)	TO (14)	TO (15)	TO (16)
3.		TO (14)	TO (14)	TO (15)	TO (15)	TO (16)	TO (17)
4.		TO (15)	TO (15)	TO (16)	TO (16)	TO (17)	TO (18)
5.		TO (16)	TO (16)	TO (17)	TO (17)	TO (18)	LP (19)
6.		TO (17)	TO (17)	TO (18)	TO (18)	LP (19)	LP (20)
7.		TO (18)	TO (18)	LP (19)	LP (19)	LP (20)	**LP (21) INT 3**
8.		LP (19)	LP (19)	LP (20)	LP (20)	LP (21)	**LP (22) INT 3**
9.		LP (20)	LP (20)	LP (21)	LP (21)	LP (22)	**LP (23) INT 3**
10.		LP (21)	LP (21)	LP (22)	LP (22)	LP (23)	LP (24)
11.		LP (22)	LP (22)	LP (23)	LP (23)	LP (24)	LP (25)
12.		LP (23)	LP (23)	LP (24)	LP (24)	LP (25)	LP (26)
13.		LP (24)	LP (24)	LP (25)	LP (25)	LP (26)	LP (27)
14.		LP (25)	LP (25)	LP (26)	LP (26)	LP (27)	LP (28)
15.		LP (26)	LP (26)	LP (27)	LP (27)	LP (28)	LP (29)
16.		LP (27)	LP (27)	LP (28)	LP (28)	LP (29)	LP (30)
17.		LP (28)	LP (28)	LP (29)	LP (29)	LP (30)	M (1)
18.		LP (29)	LP (29)	LP (30)	LP (30)	M (1)	M (2)
19.		LP (30)	LP (30)	M (1)	M (1)	M (2)	M (3)
20.		M (1)	M (1)	**M (2) INT 1**	M (2)	M (3)	M (4)
21.	M (1)	M (2)	M (2)	**M (3) INT 1**	M (3)	M (4)	M (5)
22.	M (2)	M (3)	M (3)	M (4)	M (4)	M (5)	
23.	M (3)	M (4)	M (4)	M (5)	M (5)	FP (6)	
24.	M (4)	M (5)	M (5)	FP (6)	FP (6)	FP (7)	
25.	M (5)	FP (6)	FP (6)	FP (7)	FP (7)	FP (8)	
26.	FP (6)	FP (7)	FP (7)	FP (8)	**FP (8) INT 2**	FP (9)	
27.	FP (7)	FP (8)	FP (8)	FP (9)	**FP (9) INT 2**	FP (10)	
28.	FP (8)	FP (9)	FP (9)	FP (10)	**FP (10) INT 2**	FP (11)	
29.	FP (9)	FP (10)	FP (10)	FP (11)	FP (11)	FP (12)	
30.	FP (10)	FP (11)	FP (11)	FP (12)	FP (12)	FP (13)	
31.	FP (11)		FP (12)		FP (13)	O (14)	

Int = performance measurements; FP = Follicle Phase; LT = Luteal Phase; M = Menses; TO = theoretical Ovulation

### Questionnaire

Before the performance tests, the participants were surveyed about their psychological state, using a questionnaire. For the psychological state, the Multidimensional Mood State Questionnaire (MMSQ) and menstrual symptom questionnaire (MSQ) were used [40, 41]. A total of 31 questions were asked. The psychological state was queried in the form of feeling comfortable-uncomfortable, good-bad, relaxed-tense, etc. There was a scale of 1–10 for each item, with “10” being declared as positive (e.g. feeling good, relaxed, etc.) and “1” as negative (e.g. unwell, tense, etc.). Due to the large number of questions, items were grouped into well-being, relaxation or alertness according to their commonality. The categorisation of the individual questions into the three areas of psychological status can be found in the supplementary material.

### Performance parameters

All performance tests were conducted according to NSCA guidelines [36]. Before the performance diagnostics were carried out, a familiarisation of the strength and jumping tests was done twice with all participants. Maximum strength was not tested, but primarily the technical components, for a deep squat without weight (including shoulder width position, hip fold under knee joint, verses on the floor) and the correct movements of a countermovement joump (CMJ) (starting position with extended hip and knee angle, flowing movement when bending and stretching, without arm action) and squat jump (SJ) (start in bending position, without upstroke, explosive extension of knees and hip angle, without arm action) according to the NSCA guidelines, were examined. Participants who were inexperienced in weight training were instructed on the correct execution of the movements. There was at least one week washout phase between the familiarisation phase and the diagnostic days.

After a standardised individual warm-up, two practice test jumps were performed for the SJ followed by three performance jumps. A break time of 60s was set between the jumps. The same procedure was followed for the CMJ. The jump height was measured using flight time (Optojump photocells, Microgate, Bozen, Italy). Tightening of the legs and swinging movements with the arms were prohibited for both jumps. The average of all three performance jumps was included in the analysis for both jumps.

Following the jump tests, the one repetition maximum (1-RM) was tested in the deep back squat (BS). After four warm-up sets (50% 10 reps; 70% 4-8reps, 80% 2-4reps, 90% 1-2reps of estimated 1-RM), the actual daily 1-RM was determined. A maximum of six trials were conducted in total with 4 min rest between each attempt. The weight was increased until the load could no longer be successfully managed. In addition, barbell speed was measured during all warm-up sets and maximum attempts (Vitruve sensor, virtue, Madrid, Spain). Maximum concentric velocity (MCV) was therefore measured for 50–100% (MCV50-100).

### Statistical analyses

The statistical analysis was carried out with the current version of the R statistical language. Linear mixed effects models (LME) were employed to evaluate longitudinal data [42, 43]. Initially, performance levels were defined according to Santos Jr. et al. in the back squat [37]. This was done in order to employ an additional independent variable as individual trainings state is likely to interact with the effects of the menstrual cycle. However, the classification could not be applied to SJ and CMJ. Individuals had advanced or highly advanced performance levels in 1-RM back squats but jumping performances did not correspond.

The performance variables SJ, CMJ, BS and MCV100 were combined by principal components analysis (PCA). The first principal component (PC-1) represented 59% of the total variance and served as dependent variable (“performance score”) afterwards. The performance parameters were mean-centred and scaled to unit variance before PCA.

The items of the questionnaires were combined into three components we termed “well-being”, “relaxation” and “alertness”. The individual scores (range 1–10) of the corresponding three groups of items were simply summed up.

Subsequently, the potential effects of the cycle phases were investigated using LME. Cycle phases were defined as fixed effects. Phase M was defined as an intercept and the differences between phases LFP and MLP were evaluated. The significance level was set to *p* < 0.05.

Random effects were assumed for individual intercepts, i.e. for initial performance levels, and for individual differences between cycle phases. The random effects were allowed to correlate. The correlations were not evaluated by inferential statistics but served interpretation of the possible influence of individual states on the effects of the cycle phase.

## Results

**Fig. 1:**
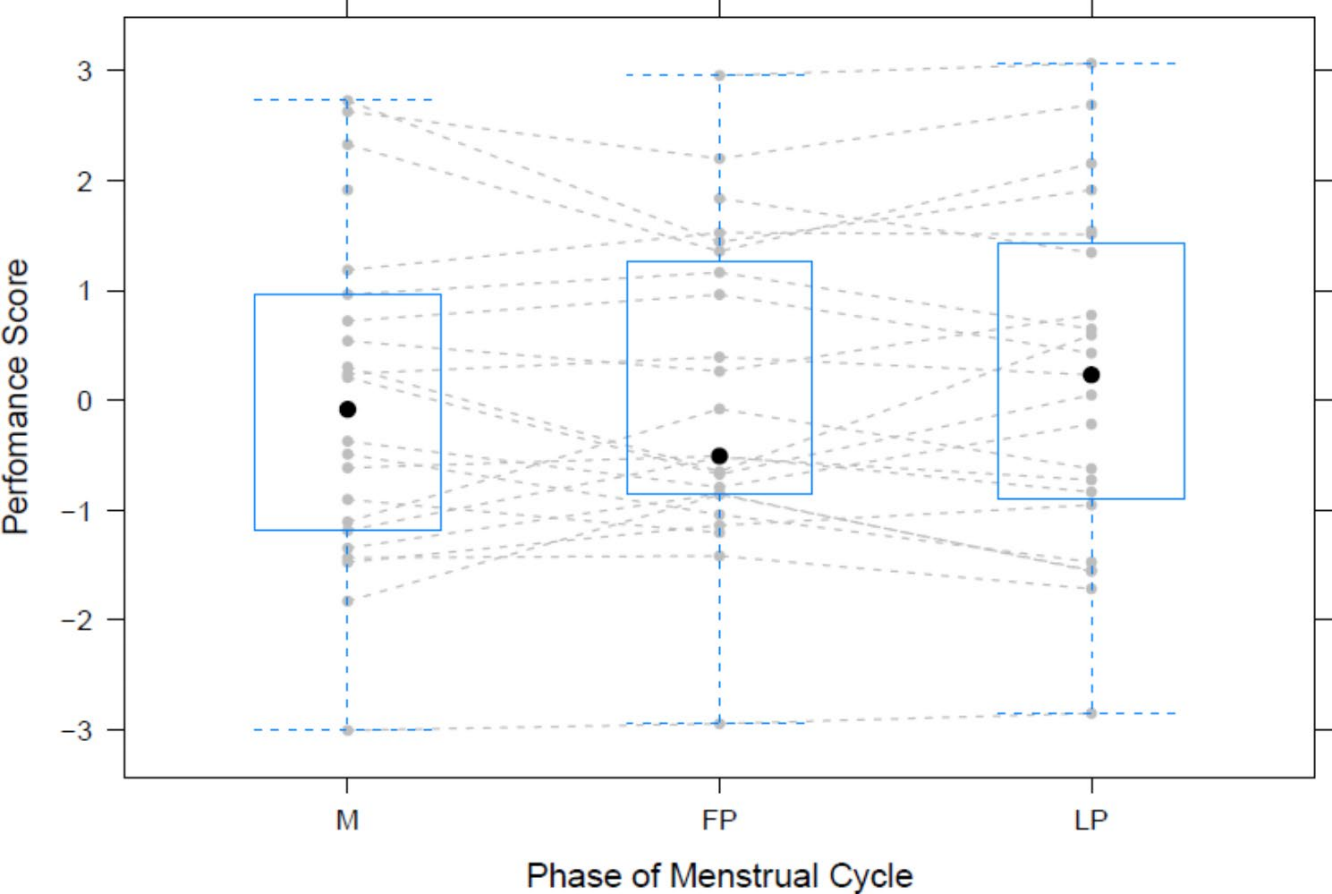
Performance score over the three phases of the menstrual cycle. The black dots indicate the mean value of all athletes, the blue squares are the mean scores with the whisker as the standard deviations. The grey lines are the individual results. FP = Follicle Phase; LP = Luteal Phase; M = Menses

Body weight, E2 and P concentration of salvia samples and raw performance data are shown in Table 2. For the performance score no significant difference was observed for the menstrual cycle phase (*p* > 0.05) (Fig. 1). The interaction of performance level and menstrual cycle phase also showed no effect on performance (*p* > 0.05). Results of the correlation analyses are shown in supplemental material.

**Table 2:** Body weight and performance data. All values are presented as mean values ± standard deviation

Parameter	Menses	Follicle Phase	Luteal Phase	
**Hormone concentration (salvia samples) (pg/ml)**				
Estradiol	0.94 ± 0.63	1.07 ± 0.94	1.03 ± 0.90	
Progesterone	12.19 ± 10.37	13.83 ± 9.55	28.42 ± 16.86	
**Body weight (kg)**				
All (*n* = 24)	67.90 ± 7.21	67.62 ± 7.02	67.54 ± 7.41	
Highly advanced (*n* = 5)*	66.03 ± 5.45	65.25 ± 5.98	63.50 ± 6.53	
Advanced (*n* = 6)*	65.95 ± 6.94	64.92 ± 6.63	65.75 ± 6.30	
Intermediate (*n* = 13)*	69.18 ± 7.24	69.54 ± 6.74	70.09 ± 7.41	
**1-RM squat (kg)**				
All (*n* = 24)	64.02 ± 11.7	65.91 ± 14.40	67.83 ± 14.10	
Highly advanced (*n* = 5)*	81.67 ± 2.36	87.00 ± 8.12	87.00 ± 6.96	
Advanced (*n* = 6)*	69.42 ± 10.23	70.30 ± 11.06	71.17 ± 10.01	
Intermediate (*n* = 13)*	57.46 ± 7.64	56.12 ± 7.25	58.17 ± 7.49	
**1-RM squat (% per BW)**				
All (*n* = 24)	0.95 ± 0.18	0.99 ± 0.24	1.02 ± 0.24	
Highly advanced (*n* = 5)*	1.24 ± 0.07	1.34 ± 0.12	1.38 ± 0.10	
Advanced (*n* = 6)*	1.05 ± 0.09	1.09 ± 0.16	1.08 ± 0.09	
Intermediate (*n* = 13)*	0.84 ± 0.13	0.81 ± 0.11	0.83 ± 0.11	
**Squat Jump (cm)**				
All (*n* = 24)	23.68 ± 6.19	23.30 ± 6.18	24.31 ± 5.94	
Highly advanced (*n* = 5)*	29.99 ± 4.02	29.07 ± 3.54	29.96 ± 2.97	
Advanced (*n* = 6)*	26.22 ± 6.27	24.34 ± 5.79	26.32 ± 4.72	
Intermediate (*n* = 13)*	21.73 ± 5.14	22.31 ± 6.43	22.66 ± 6.05	
**Countermovement jump (cm)**				
All (*n* = 24)*	27.02 ± 7.37	27.25 ± 7.64	27.90 ± 7.49	
Highly advanced (*n* = 5)*	34.71 ± 5.57	35.19 ± 4.60	34.61 ± 3.63	
Advanced (*n* = 6)*	31.32 ± 7.27	28.27 ± 7.42	30.87 ± 6.32	
Intermediate (*n* = 13)*	21.51 ± 8.04	24.12 ± 9.63	25.11 ± 7.10	

* Classification based on the performance level of Santos et al. [37]

### Questionnaire

**Fig. 2:**
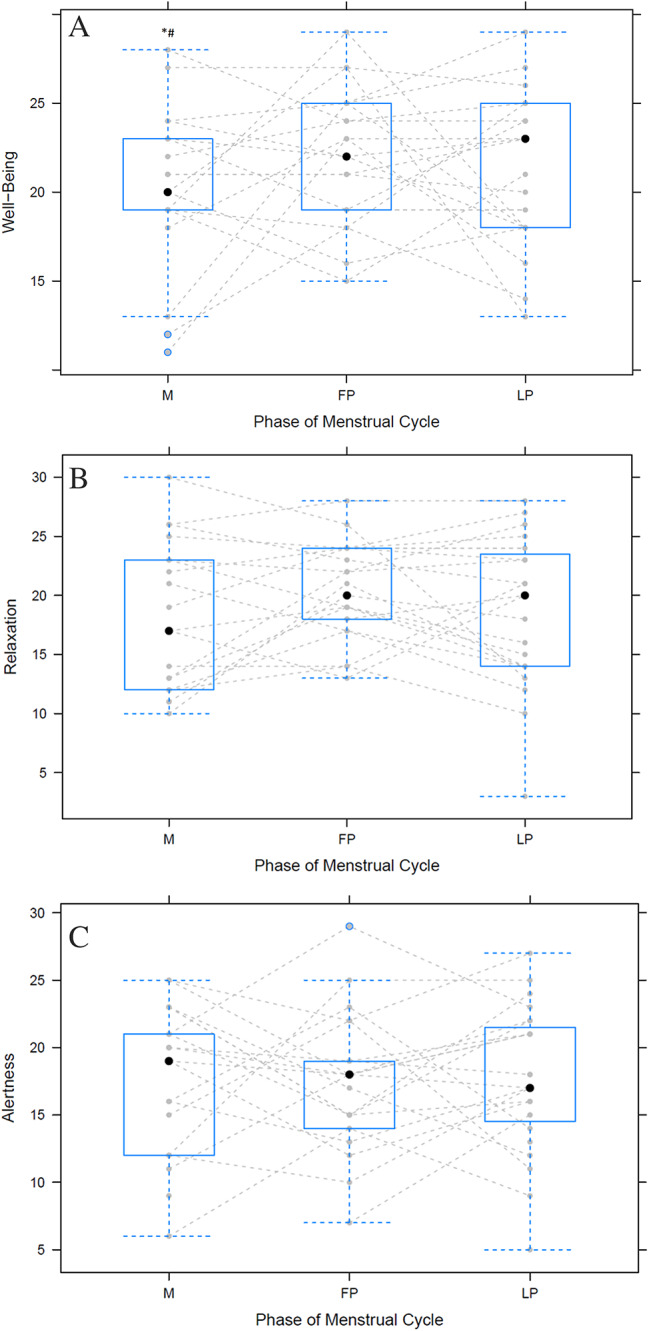
Well-being (**A**), Relaxation (**B**) and Alertness (**C**) score over the three phases of the menstrual cycle. The black dots indicate the mean value of all athletes, the blue squares are the mean score with the whisker as the standard deviations. The grey lines are the individual results. * indicates significant difference to FP; # indicates significant difference to LP. FP = Follicle Phase; LP = Luteal Phase; M = Menses

For well-being, a significant lower score was detected for M to FP (*p* < 0.05) and LP (*p* < 0.05). No significant difference was observed between FP and LP (*p* > 0.05) and no effect of the performance level was detected (*p* > 0.05) (Fig. 2A).

For the Relaxation and Alertness score, no significant difference was found (*p* > 0.05) between the cycle phase and the performance level (*p* > 0.05) (Fig. 2B and C).

## Discussion

This study aimed to investigate the influence of the MC on maximal back squat performance and jumping ability assessed by SJ and CMJ according to the performance level of the athletes. In addition, psychological state during the intervention days was surveyed. No significant differences were found between the MC phases in performance scores and between performance levels. Only a significant difference in well-being between the phases could be observed. During M the well-being was distinguished compared to the other two phases.

Studies on the effects of the menstrual cycle on performance are limited and the current studies are of low quality [8–10]. Nevertheless, a few reviews have emerged in recent years [8–10, 44, 45]. If the individual-included studies are considered, only limited studies have investigated practical and relevant endpoints [8]. Additionally, these have tended to focus on jumping performance rather than on maximal strength, primarily different jumping tests were performed [8]. In terms of practical strength capacity, only one study was conducted and used a Smith-Machine for back squats [17]. However, neither a full squat nor maximal strength test was performed. Therefore, this study is the first to investigate the influence of MC on dynamic maximum barbell back squat performance. In addition, unlike the majority of previous research, more than two cycle phases were considered, and the intervention days were defined with two independent methods (cycle diary and hormone analyses) [8]. Although, no significant differences were found for the performance score, the classification of performance level by Santos et al. suggests that there may be an influence on the strength ability depending on the performance level. The absolute values for the squat (Table 2) and the correlation analyses for the absolute and relative strength capacity (supplemental material) in the squat indicate that an influence of the MC might occur at a higher performance level. In highly advanced athletes, the absolute and relative strength values in the M were lower than in the other two phases.

It is well known that with increasing performance levels, performance can be affected by a variety of factors [29–31]. It could therefore be speculated that differences between the menstrual cycle phases might only occur in athletes above a certain performance threshold. However, this speculation should be taken with caution, as only a very small number of participants had this performance level in this study.

Interestingly, this phenomenon was not observed in the jumping exercises. Perhaps the different forms of loading (explosive force development without load and maximum force development against a load) produce different effects. For example, muscle-tendon stiffness plays a major role in jumping exercises but less on maximal strength performance. Research on the MC has shown a significant decrease in lower limb muscle-tendon stiffness [46], in the muscle stretch reflex [47], as well as an increase in muscle extensibility [48], joint laxity, and injury risk [49] during ovulation. Lower limb stiffness is considered important for promoting jumping and hopping activities as compliant muscles and tendons reduce the rate of force development and increase electromechanical delay [46]. Despite these observations, no difference in jumping performance could be detected between the phases. This is consistent with previous studies with different type of participants [23–25, 27]. Interestingly, the jumping ability of female soccer players in the CMJ was similar to the advanced and lower than the highly advanced athletes in this study [23]. Comparing the jumping performance of our participants with other athletes, they are below elite athletes from volleyball or track and field [50, 51]. Consequently, the performance level concerning jumping ability might be too low in this study to identify possible differences.

Regarding the results of the questionnaire, a significant difference between the phases could only be found in the category “well-being”. Well-being is significantly lower during M than in LFP and MLP. This observation is consistent with previous studies [9, 32–35]. In elite sports, the psychological component plays a crucial role in addition to the physiological one [34, 44]. If an athlete feels uncomfortable due to menstrual symptoms, this could affect performance [35]. Despite these not being able to confirm these differences statistically, due to the marginal changes, it is important to note that these could have significant effects in elite sports. In many sports where a few centimetres or hundredths of a second are at stake, even slight changes can have a marked effect on performance.

Based on the new findings and the limiting data on dynamic maximum strength exercises relevant to practice, further investigations are urgently needed regarding performance where the performance level of the athlete is taken into consideration. This is especially the case with very well-trained or elite athletes as it could be that it is only at these higher performance levels that possible differences can be identified.

### Limitations

In addition to the new findings presented it is important to note that, this study also has some limitations. One is the determination of the hormone concentrations of E2 and P as well as the identification of ovulation by determining LH to separate FP from LP. Even if there is a lively discussion about the rises and rhythms of hormones, no absolute values can be given as a reference for women. Hormone concentrations vary, not only between individuals but also between cycles in different women. Therefore, this study deliberately used only an indirect method to identify the phases. With the assistance of the cycle diary, the length of the bleeding phase and the increase in progesterone concentration in the LP, the three different phases could be determined very precisely. In addition, cycle days were explicitly chosen which did not lie in the transition phase of two cycle phases. Even though the highest hormone concentrations may not be present on the intervention days, the classification into the three cycle phases M, LFP, MLP could be carried out. Another limitation is the low number of highly advanced female athletes in the squat and jumps exercises. Consequently, in future investigations explicit attention should be paid to the performance level and ideally highly trained athletes in their sports should be recruited.

## Conclusion

This study investigated the influence of the menstrual cycle on dynamic maximum squat performance, jumping ability and psychological state considering the level of performance. No differences were detected in terms of a power performance score. However, if the performance level and the individual performance parameters are considered, the first indications were found that differences in strength capacity can occur at higher performance levels. During the M, highly advanced trained women have a lower strength capacity than in the LFP or MLP. Besides in well-being a lower score was detected in M compared to the other two phases. However, the results should not be overinterpreted due to the small sample size in the individual performance levels. In addition, no statement can be made about elite female strength athletes. Further studies should be carried out in this area and different performance levels should be analysed.

### Main outcomes (Key points)

- There are hardly any studies on the influence of the menstrual cycle on practical exercises and even fewer that take into consideration the performance level of the athlete.
- Across all data, the menstrual cycle does not appear to influence power performance.
- There are indications of differences in strength capacity depending on the performance level, but no clear conclusions can currently be made.
- The well-being of women is influenced by the cycle phases. During menstruation, the participants feel less comfortable than in the other two phases.

## Electronic supplementary material

Below is the link to the electronic supplementary material.

Supplementary Material 1

Supplementary Material 2

## Data Availability

The data presented in this study are available on request from the corresponding author.
