# Supplementary material for: The effect of the menstrual cycle phases on back squat performance, jumping ability and psychological state in women according to their level of performance -a randomized three-arm crossover study
Source: BMC Sports Sci Med Rehabil. 2024 Nov 2;16:224. doi: 10.1186/s13102-024-01010-4 (PMC11531699; doi:10.1186/s13102-024-01010-4)
Supplement: Supplementary file 2 — Supplementary Material 2 [file 13102_2024_1010_MOESM2_ESM.docx]

**Supplemental Material:**

Categorization of psychological state questions to the categories well-being, relaxation and alertness.

Well-being:

MMSQ: Unhappy-Happy; Restless-Calm; Poor-Good; Uncomfortable-Comfortable

MSQ: Headaches; Dizziness, Depression; Confusion; Weight gain; Swollen extremities; Breast tensions; Inflated; Lower abdomen pain; Back pain; General pain

Relaxation:

MMSQ: Tense-Relaxed; Nervous-Balanced

MSQ: Mood fluctuation; Irritability; anxious; Tension; Heart palpitations; Craving for sweets, Increased appetite; Crying

Altertness:

MMSQ: Tired-Wake; Energy-poor-Energy-rich; Exhausted-Rested

MSQ: Fatigue; Forgetfulness; Sleep disorders

Correlation analyses

Table S1: Estimates, standard errors, t-statistics and p-values of the linear mixed effects model fitted to the back squat performances [kg]. M: menses (model intercept), FP: follicle phase; LP: luteal phase.

|  | Value | Std.Error | DF | t-value | p-value |
| --- | --- | --- | --- | --- | --- |
| **(Intercept)** | 66.0 | 2.74 | 42 | 24.1 | <0.0001 |
| **FP** | -0.02 | 0.973 | 42 | -0.02 | 0.984 |
| **LP** | +1.49 | 0.762 | 42 | 1.96 | **0.0571** |

Table S2: Random effects correlations of the of the model shown in table. 1.

|  | (Intercept) | FP | LP |
| --- | --- | --- | --- |
| **(Intercept)** | 1 |  |  |
| **FP** | 0.264 | 1 |  |
| **LP** | 0.455 | **0.932** | 1 |

Table S3: Estimates, standard errors, t-statistics and p-values of the linear mixed effects model fitted to relative back squat performances. M: menses (model intercept), FP: follicle phase; LP: luteal phase.

|  | Value | Std.Error | DF | t-value | p-value |
| --- | --- | --- | --- | --- | --- |
| **(Intercept)** | 98.8 | 4.5 | 42 | 21.9 | <0.0001 |
| **FP** | +0.18 | 1.5 | 42 | 0.07 | 0.948 |
| **LP** | +2.69 | 1.2 | 42 | 2.29 | **0.027** |

Table S4: Random effects correlations of the of the model shown in table 3.

|  | (Intercept) | FP | LP |
| --- | --- | --- | --- |
| **(Intercept)** | 1 |  |  |
| **FP** | 0.317 | 1 |  |
| **LP** | 0.686 | **0.862** | 1 |

Table S5: Estimates, standard errors, t-statistics and p-values of the linear mixed effects model fitted to counter movement jump performances [cm]. M: menses (model intercept), FP: follicle phase; LP: luteal phase.

|  | Value | Std.Error | DF | t-value | p-value |
| --- | --- | --- | --- | --- | --- |
| **(Intercept)** | 29.1 | 1.59 | 42 | 18.3 | <0.0001 |
| **FP** | +0.123 | 0.522 | 42 | 0.235 | 0.816 |
| **LP** | -0.549 | 0.539 | 42 | -1.02 | 0.314 |

Table S6: Random effects correlations of the of the model shown in table 5.

|  | (Intercept) | FP | LP |
| --- | --- | --- | --- |
| **(Intercept)** | 1 |  |  |
| **FP** | -0.023 | 1 |  |
| **LP** | -0.05 | 0.217 | 1 |

Table S7: Estimates, standard errors, t-statistics and p-values of the linear mixed effects model fitted to squat jump performances [cm]. M: menses (model intercept), FP: follicle phase; LP: luteal phase.

|  | Value | Std.Error | DF | t-value | p-value |
| --- | --- | --- | --- | --- | --- |
| **(Intercept)** | 25.1 | 1.39 | 42 | 18.1 | <0.0001 |
| **FP** | -0.673 | 0.47 | 42 | -1.43 | 0.16 |
| **LP** | -0.138 | 0.41 | 42 | -0.334 | 0.74 |

Table S8: Random effects correlations of the of the model shown in table 7.

|  | (Intercept) | FP | LP |
| --- | --- | --- | --- |
| **(Intercept)** | 1 |  |  |
| **FP** | -0.02 | 1 |  |
| **LP** | -0.05 | 0.217 | 1 |
